# Oligodendroglial Lineage Cells in Thyroid Hormone-Deprived Conditions

**DOI:** 10.1155/2019/5496891

**Published:** 2019-04-30

**Authors:** Min Joung Kim, Steven Petratos

**Affiliations:** Department of Neuroscience, Central Clinical School, Monash University, Prahran, Victoria 3004, Australia

## Abstract

Oligodendrocytes are supporting glial cells that ensure the metabolism and homeostasis of neurons with specific synaptic axoglial interactions in the central nervous system. These require key myelinating glial trophic signals important for growth and metabolism. Thyroid hormone (TH) is one such trophic signal that regulates oligodendrocyte maturation, myelination, and oligodendroglial synaptic dynamics via either genomic or nongenomic pathways. The intracellular and extracellular transport of TH is facilitated by a specific transmembrane transporter known as the monocarboxylate transporter 8 (MCT8). Dysfunction of the MCT8 due to mutation, inhibition, or downregulation during brain development leads to inherited hypomyelination, which manifests as psychomotor retardation in the X-linked inherited Allan-Herndon-Dudley syndrome (AHDS). In particular, oligodendroglial-specific MCT8 deficiency may restrict the intracellular T_3_ availability, culminating in deficient metabolic communication between the oligodendrocytes and the neurons they ensheath, potentially promulgating neurodegenerative adult diseases such as multiple sclerosis (MS). Based on the therapeutic effects exhibited by TH in various preclinical studies, particularly related to its remyelinating potential, TH has now entered the initial stages of a clinical trial to test the therapeutic efficacy in relapsing-remitting MS patients (NCT02506751). However, TH analogs, such as DITPA or Triac, may well serve as future therapeutic options to rescue mature oligodendrocytes and/or promote oligodendrocyte precursor cell differentiation in an environment of MCT8 deficiency within the CNS. This review outlines the therapeutic strategies to overcome the differentiation blockade of oligodendrocyte precursors and maintain mature axoglial interactions in TH-deprived conditions.

## 1. Introduction

The central nervous system (CNS) coordinates all vital and higher-order functions through its integrated network of neurons supported by the glial cells that cooperate to maintain the integrity of neurological function. Oligodendrocytes (OLs) play a fundamental supportive role to the axonal processes of neurons through the insulating myelin membranous sheath. However, the loss of these cells or specific developmental defects during oligodendrogenesis results in the denudement of axons, potentiating the brain's vulnerability to further neurodegeneration [1–3]. More specifically, OLs support the integrity of the CNS neurons and their axons, structurally and metabolically. The failure of this support leads to damaged axons and impaired myelin ensheathment, resulting in latency in electrical propagation between neurons. In multiple sclerosis (MS), demyelinated axons can deteriorate over time due to the failure in spontaneous remyelination. The reason for this failure is that stalled differentiation of oligodendrocyte precursor cells (OPCs) is a common fate for these cells around demyelinated lesions [4]. A lack of trophic support to OPCs, such as the potent differentiation hormone, thyroid hormone (TH), may lead to limited oligodendroglial differentiation. Here, this review focuses on the role of OLs in protecting the integrity of axons and the dynamic axoglial unit, providing novel insights into how we may be able to overcome the differentiation blockade of OPCs under TH-deprived conditions.

## 2. Role of Oligodendrocytes in Protecting the Integrity of Axons and Axoglial Unit

Oligodendrocytes (OLs) are abundant macroglial cells that during postnatal development ensheath nude axons with its extensive protective plasma membrane, consisting of predominantly lipid (approximately 70%) and glycoproteins known as myelin that is dynamically remodeled in childhood, adolescence, and even in adulthood [5]. Mature OLs are characterized by their multipolar morphology with extensive processes that can produce myelin membrane lamellae, along with robust expression of mature OLs and myelin markers such as proteolipid protein (PLP), myelin basic protein (MBP), myelin oligodendrocyte glycoprotein (MOG), 2′,3′-cyclic nucleotide 3′-phosphodiesterase (CNPase), and myelin-associated glycoprotein (MAG) [6].

The highly organized microstructure of the glial cell and the axonal process of the neuron it supports, termed the axoglial junction, include the segregated array of distinct molecular and functional domains that enable the rapid propagation of action potentials [7]. These domains include the microanatomical paranode, juxtaparanode, and internode, which are physiologically important for maximizing the transmission of action potentials [7]. Lamellated compact myelin membrane extends at variable internodal lengths dependent on the fascicle interrupted by discrete regions of axolemma known as the node of Ranvier [8]. Voltage-gated sodium channels that are concentrated at the node of Ranvier are mainly responsible for the axonal depolarization that is required for the generation of action potentials [8]. Myelin sheaths provide fast propagation of electrical signals by reducing axolemmal capacitance, protecting axons from the leakage of ions and thereby potentiating saltatory nerve conduction within millisecond response times. This physiological property facilitates the communication between integrated neural circuits for the execution of complex physiological responses [9, 10].

The axoglial unit consists of the molecular complex of neurofascin 155 (NF155), axonal Caspr1 (contactin-associated protein 1), and contactin. Neurofascin 155 (NF155) is a cell adhesion molecule of the L1 subgroup of the immunoglobulin G superfamily, which is involved in neurite outgrowth, fasciculation, and interneuronal adhesion [11–13]. It has been shown that NF155 is expressed on paranodal myelin membranes, is a glial cell adhesion molecule of the paranodal junctional complex, and is restricted to the paranodal loops of the sheath, where they interact with Caspr on the axon [14]. Caspr1 is encoded by the *cntnap1* allele, and the product is expressed as a transmembrane protein on the paranodal axolemma. Caspr belongs to the neurexin superfamily of molecules and has intra- and extracellular binding motifs for protein-protein and protein-carbohydrate interactions [15–17]. The paranodal expression of NF155 and Caspr1 is critical for axoglial communication. Loss-of-function mutations in these molecules may cause a significant neurological dysfunction in the central and peripheral nervous system, as exhibited in a NF155-knockout mouse, identified delay in postnatal synapse elimination at the neuromuscular junction [18]. This paranodal molecular dysfunction can alter the conductive properties of myelinated axons, causing neuromuscular junction blockade.

Furthermore, a recent finding of individuals diagnosed with congenital hypomyelinating neuropathy, with a dominant negative mutation in the *CNTNAP1* gene, demonstrated a widening of the paranodal junctional gap between the loops and axolemma along with thinly myelinated axons [19, 20]. Another molecule, contactin, is encoded by the *cntn-1* gene, a glycosylphosphatidylinositol- (GPI-) linked membrane glycoprotein which has been identified as a critical signal for axoglial communication in the CNS myelin. A *cntn-1*-knockout mouse model has shown an apparent hypomyelination phenotype in the optic nerve, cerebellum and corpus callosum, and defective myelin development [21]. Moreover, the transient delay in OL development and the disrupted paranodal junction were also observed in this mutant model [21]. Collectively, these data indicate the importance of orchestrating these subdomains to potentiate the interaction of these molecules within the axoglial units.

The neuroaxonal supportive role of the myelin-ensheathing membrane derived from mature OLs can extend beyond the propagation of action potentials to include metabolic support of highly functional neurons. Neurons have the most substantial metabolic demands and so their highly active axons are vulnerable under conditions of increased electrical activity [22]. In fact, the failure of structural and metabolic support of neurons may lead to axonal damage and impaired myelin ensheathment, resulting in delayed signaling at synapses, thereby potentiating the brain's vulnerability to overt neurodegeneration.

Defective myelin protein expression has been clearly shown to lead to significant neurological dysfunction as manifested in gain- or loss-of-function mutations identified within animal models and human diseases. For example, the duplication of *plp1* in mice leads to a dysmyelinating phenotype, observed in Pelizaeus-Merzbacher disease (PMD) [23]. Additionally, within the *mbp* knockout (*mbp^−/−^*) mouse model, which displays a shiverer phenotype, exhibits a significant hypomyelination with only mild axonal swelling in spinal cord white matter [24, 25]. *In vivo* studies of these myelin protein mutant mouse models clearly support the structural role supplied to neurons by mature OLs. However, within the PLP-DM20-deficient mice, axonal swelling and degeneration have been observed in the absence of myelin abnormalities [26, 27].

Failure of oligodendroglial support of axons results in severe neurological disorders, as neurons become vulnerable for further neurodegeneration which manifest in the acquired demyelinating diseases such as multiple sclerosis (MS) or during inherited forms of leukodystrophy [28]. Indeed, preservation of OLs and their myelin sheaths would promote neuroprotection and limit permanent neurological deficit. Moreover, achieving CNS repair through remyelination would be a viable option if the diseased tissue environment is modified; the major limitation in this disease tissue milieu is the stalled maturation of endogenous OPCs and the number of cells available to effectively perform the repair [29], partially due to lack of trophic support or differentiation signals [30].

## 3. Thyroid Hormone Signaling during Oligodendrocyte Development

Thyroid hormones (TH) are fundamental to brain development, playing a chief role in regulating cell migration and differentiation, synaptogenesis, and eventually myelination (for review, see [31]). The thyroid gland physiologically produces predominantly the prohormone thyroxine (T_4_) and only a small amount of active hormone, triiodothyronine (T_3_). The primary effector cellular response occurs once T_3_ binds to its receptors, TH receptor alpha and beta (TR*α* and TR*β*) in the nucleus, activating the TH response element to initiate transcription [32]. Consequently, the concentration of intracellular T_3_ is mostly dependent on the activity of the plasma membrane-localized transporters that can facilitate the uptake of extracellular T_4_ and T_3_ to eventuate a cellular response at the transcription and translation level [33].

### 3.1. The Role of Thyroid Hormone during Oligodendrocyte Development

Significant evidence supports the mitogenic role of TH during OL development [34–38]. The effects of TH on OPC proliferation and differentiation depend on the cells' specific stage of development [39–41]. The precise timing of T_3_ stimulation of OPCs can modulate their replication, survival, and myelin production upon maturation into OLs that are all developmentally regulated events critical for integrated brain function [42]. T_3_ induces cell cycle exit of OPCs along with platelet-derived growth factor (PDGF), by downregulating the gene encoding TR*α* and upregulating TR*β*1, which then leads to their terminal differentiation [43, 44]. The morphological and functional maturation of OLs is stimulated by T_3_ to upregulate myelin gene expression, including myelin basic protein (MBP) and proteolipid protein (PLP) [45]. The consistent mRNA level of these myelin proteins is significantly reduced in the hypothyroid neonatal rat brain, emphasizing the role of T_3_ during myelin gene production [46, 47]. This physiological developmental switch is placed in context when observing OPCs under conditions whereby these cells are subjected to acute and chronic deprivation of T_3_ in culture, generating higher numbers of preoligodendrocytes and corresponding with lower numbers of mature OLs [41]. Furthermore, these investigators also showed that a lack of cytoplasm-filled myelin membrane was evident in T_3_-deficient cultures, suggesting the role of T_3_ in the later stages of OL maturation and formation of myelin. In particular, the altered distribution of MBP during OL differentiation would also contribute to the failure of myelin compaction in the hypothyroid animals [41], whereas cultures derived from hyperthyroid rats have shown OLs with longer processes compared to the shorter processes in the OLs derived from hypothyroid rats [48]. Collectively, these data suggest that T_3_ is a vital mitogen during OL development, regulating cell cycle events, differentiation, and maturation through the formation of complex myelin membrane.

### 3.2. The Role of Thyroid Hormone in Regulating Mitochondrial Activity for Oligodendrocyte Development and Myelin Biogenesis

The neurobiological effects of TH are orchestrated via specific genomic and nongenomic pathways [31, 49]. The genomic transcriptional events occur once T_3_ binds to TH receptors (TRs) *α* and *β* that are transcribed from *THRA* and *THRB*, respectively, in the nucleus [32, 49]. TR*α*1, TR*β*1, and TR*β*2 are the most thoroughly investigated isoforms whereas the TR*α*2 and TR*α*3 isoforms do not bind TH [50–53]. Resistance to thyroid hormone (RTH) occurs due to mutations in the TR*α* and TR*β* genes, whereby individuals heterozygous for the dominant negative forms of the translated receptors exhibit neurological and developmental psychiatric abnormalities [54]. It is now clear that metabolically active OLs play pivotal roles in neurocognition and plasticity [2]. Dysfunction in oligodendrogenesis and mature OL plasticity has been reported in mouse models of cognitive decline or in genetic and acquired human neurodevelopmental abnormalities [55]. Fundamental to these abnormalities is the dynamic health of the mature and developing OL capable of forming the myelin membrane upon demand, orchestrated by the activity-dependent mechanism of the neuron that it ensheaths [55]. This neuroglial coupling requires substantial metabolic reinforcement due to the constant energy demands of this union [55]. It is the oligodendroglial cell mitochondria that drive their myelination capacity only when metabolic parameters are conducive, and failure in the mitochondrial TCA cycle (requiring T_3_ signaling) during acquired demyelinating or genetic hypomyelinating disease limits the capacity to generate myelin membrane [56]. However, recent data has implicated that a T_3_-deficient window maybe necessary to drive OPC proliferation from the subventricular zone in a mouse model of demyelination [57]. Whether this evidence is at all relevant to what occurs in the chronic MS lesion remains to be elucidated.

Both nongenomic and genomic outcomes of TH influence mitochondrial physiology, where the majority of cellular adenosine triphosphate (ATP) is generated (for review, see [58]). Low TH levels are known to impair mitochondrial energy production, as a chronic mitochondrial deficiency can lead to chronic fatigue due to hypothyroidism [59]. Moreover, the mitochondrial dysfunction and subsequent energy deficits profoundly impact the high-energy demand processes of the CNS, including myelin formation and plasticity [60, 61]. For instance, increases in mitochondrial activity have been shown to correspond with the higher metabolic demand in dysmyelinated axons, as demonstrated by cytochrome *c* histochemistry in the *shiverer* dysmyelination mouse model [62]. Furthermore, the intra-axonal mitochondrial density is significantly increased in demyelinated axons of *plp1*-overexpressing mice, demonstrating profound energy uncoupling with attempts at compensation by the neuron in this model of the Pelizaeus-Merzbacher disease, a human inherited leukodystrophy that exhibit hypomyelination and subsequent neurodegeneration [63]. These studies emphasize the importance of bidirectional movement of metabolic substrates from OLs to neurons that can include TH, enabling the physiological maintenance of electrical propagation driven by mitochondrial activity. Failure of this axoglial metabolic transport system may lead to the neurodegenerative changes governing cognitive decline and progression in myelin-related disorders, as occurs in MS progression.

## 4. Transporters of Thyroid Hormones in the Central Nervous System

The passage of THs across the plasma membrane is facilitated by its transporters that include the family of solute-like carrier (SLC) proteins known as the monocarboxylate transporters (MCT8 and MCT10, encoded by the SLC16A2 and SLC16A10 genes, respectively), organic anion-transporting polypeptide (OATP1C1 or SLC01C1), and L-type amino acid transporter (LAT1 and LAT2, also known as SLC7A5 and SLC7A8, respectively) [64, 65]. However, these transporters have varying affinities for THs and display variable distribution patterns in tissues, with OATP1C1 and LATs transporting amino acid substrates, being a secondary transporter of THs [65]. The only substrate identified for MCT8, however, is T_3_, highlighting this transporter's fundamental importance in the cellular physiology of TH regulation [66].

Monocarboxylate transporter 8 (MCT8) is encoded by the gene *slc16a2* located on the long arm (q) of the X chromosome at position 13.2 (Xq13.2) in humans (gene ID: 6567) [67]. MCT8 was first identified from the DNA library screen of the rat brain and by using functional studies in *Xenopus laevis* oocytes [66]. It has been identified that MCT8 is highly expressed in neuronal populations of the cerebral and cerebellar cortex, hippocampus, striatum, and hypothalamus [68, 69]. The critical role of MCT8 within humans was recognized retrospectively by identifying mutations of the *SLC16A2* gene (that encodes MCT8) producing the phenotype of severe psychomotor retardation, defined as Allan-Herndon-Dudley syndrome (AHDS) [70]. The hypomyelinating phenotype of these patients suggests functional mutations in MCT8 may affect the development of OLs and myelination similar to other severe inherited leukodystrophies such as Pelizaeus-Merzbacher disease (PMD) caused by the duplication of the PLP1 gene. These conditions display unstable hypomyelination with defects in OL differentiation identified in humans and in their respective models of disease [26, 71–74]. Additionally, the overexpression of the *PLP1* gene leads to apoptosis of OLs, and the absence of the *PLP1* gene leads to impaired axonal transport promulgating neurodegeneration and disability [73, 75–81].

It has been understood that MCT10 is the secondary transporter of T_3_ as it favors the plasma membrane transport of aromatic amino acids, performing its common T-type amino acid transporter (TAT) function [82, 83]. However, MCT10 may be a better facilitator of T_3_ uptake than MCT8 [83], even though MCT8 is highly homologous to MCT10 as they share 49% of amino acid sequences and MCT8 and MCT10 can form heterodimers [66]. MCT10 shows overlapping expression with MCT8 and has recently been identified as an important transporter expressed within developed white matter tracts of the mouse brain [84], which may suggest a functional role in mature oligodendrocytes. The expression of MCT10 has also been previously reported to be enriched postnatal microglial emphasizing an important role in these endogenous monocytic cells [85]. However, its major role in brain development is yet to be established, since MCT10 knockout mice do not display a neurological phenotype and the observations reported in MCT10 and MCT8 double knockout mice suggest that MCT10 can normalize the brain-specific hypothyroid status (T_4_ levels) exhibited in MCT8 knockout mice alone [86]. The role of MCT10 in oligodendrogenesis is even more obscure, without thorough investigation.

## 5. A Novel Role for Monocarboxylate Transporter 8 in Oligodendrocyte Maturation

The expression of MCT8 has been identified on neurons and astrocytes [68, 69]. However, MCT8 localization on OL plasma membranes has not been elucidated thoroughly. We have recently defined the MCT8 expression and function on mature human OLs derived from human embryonic stem cells (hESC) [87]. Furthermore, a novel role of the T_3_-specific transporter, MCT8 in OL maturation and myelination, has been suggested [87]. As a corollary, the role of oligodendroglial cell-specific MCT8 will also be discussed below in the context of axoglial integrity.

### 5.1. Dysfunction of MCT8 Inducing Neurological Disease: Allan-Herndon-Dudley Syndrome (AHDS)

As discussed, mutation of the *SLC16A2* gene (encoding MCT8) is pathogenic in the *X-linked* inherited dysmyelinating disorder, AHDS. The original clinical description of this psychomotor retardation disorder was observed in 1944 by William Allan, Nash Herndon, and Florence Dudley (Allan et al., 1944). Through the case studies of affected individuals, investigators have found that the development of myelin is substantially delayed culminating in severe brain damage in all male offspring along with abnormal TH parameters in the periphery [88–94]. Patients exhibiting MCT8 mutations present with severe neurological symptoms including hypotonia, muscular hypoplasia, and developmental retardation [88–94] and severe cognitive impairment, a classical outcome of inappropriate myelin formation [88, 89]. The cognitive impairment observed in the patients with AHDS initiated the preliminary investigations of the role of MCT8 in supplying T_3_ to the brain [88, 89]. The elevated levels of serum T_3_ in the patients with AHDS produce a hyperthyroid state in peripheral tissues; however, a hypothyroid state exists within the CNS due to the diminished T_3_ levels within the brain, a consequence of deficient transport across the blood brain barrier [95]. Postmortem analysis performed on an 11-year-old AHDS boy has revealed prominent hypomyelination by MBP immunostaining [96], strongly suggesting that the availability of T_3_ within the CNS may be abrogated specifically in OLs due to the lack of functional MCT8, thereby limiting the differentiation capacity of this cell lineage, causing dysmyelination during brain development. Although myelination has been reported in longitudinal studies of AHDS patients, the developmental myelination is incomplete and neurological phenotypes persist [97–99]. These clinical and histological findings suggest MCT8 is a key membrane transporter for the intercellular delivery of T_3_ particularly during OPC development, thereby promoting OL maturation and the development of myelin [100].

### 5.2. A Role for MCT8 in Axoglial Integrity

**Figure 1:**
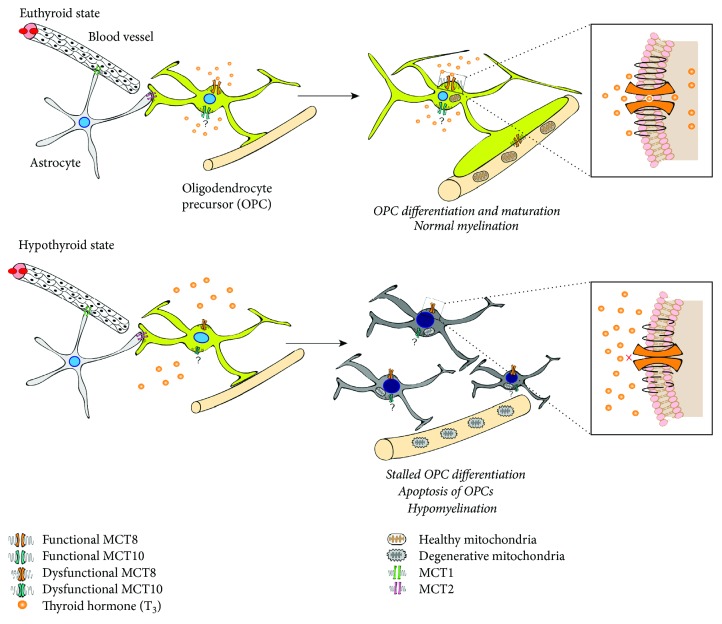
Hypothyroid state within the CNS leads to cellular hypothyroidism in oligodendrocytes. In the euthyroid state, functional MCT8 expressed on oligodendrocyte precursors (OPCs) is able to transport thyroid hormone (T_3_) across the plasma membrane promoting their differentiation and maturation. Normal myelination subsequently occurs. When MCT8 is dysfunctional due to various points or frameshift mutations, or in the context of neuroinflammation, intracellular T_3_ transport is impeded, as a result of dysfunctional MCT8. This results in a profound hypothyroid state in OPCs, leading to their stalled differentiation, or indeed apoptosis, with the eventual neurobiological result being hypomyelination and the clinical outcome being neurodegeneration and cognitive decline.

MCT8 is highly expressed in neuronal populations of the cerebral and cerebellar cortex, hippocampus, striatum, and hypothalamus [68, 69]. It is likely that during disease, different subpopulations of neural cell types which express transporters in different proportions are differentially affected by a lack of MCT8 either acutely or through protracted chronicity [101]. In order to explore the pathogenic mechanisms of AHDS, MCT8-knockout (MCT8-KO) mice were generated [102]. Although MCT8-KO mice were able to replicate abnormal peripheral TH levels, leading to systemic hyperthyroidism, these mice do not display the equivalent neurological and behavioral abnormalities exhibited in AHDS patients [103, 104]. This may suggest that there exists a compensatory mechanism in the mouse brain without functional MCT8. Indeed, this was demonstrated in MCT8-OATP1C1-double knockout mice [105]. These double knockout mice exhibited elevated T_3_ and reduced T_4_ levels in serum whereas the uptake of T_3_ and T_4_ was significantly reduced in the brain. Furthermore, deiodinase activities and T_3_-regulated target gene expression including *Hr*, *RC3*, and *Aldh1a1* were substantially decreased [105]. Moreover, the effect of limited functional MCT8 on myelin formation was investigated in a developmental zebrafish model where *slc16a2* was deleted using zinc-finger nuclease- (ZFN-) mediated targeted gene editing [106]. Unlike *slc16a2-*KO mice, these zebrafishes exhibited neurological and behavioral deficiencies similar to AHDS patients. These data suggest that MCT8-dependent T_3_ transport is required for normal OL development and maturation (as illustrated in Figure 1). In line with this, our group has shown that in human differentiating oligodendroglial cells derived from hESCs, an increase in apoptosis along with an abrogated myelinating capacity occurs upon acute knockdown of MCT8, suggesting that MCT8 plays a central role in oligodendroglial cell differentiation [87].

The importance of central TH metabolism is highlighted in patients with monogenic mutations such as those occurring in AHDS that exhibit elevated peripheral free T_3_ and either normal or reduced T4 levels [88, 89]. However, even in euthyroid individuals, tissue hypothyroidism can promulgate a significant morbidity and even mortality as a consequence to altered tissue-specific TH metabolism [107]. Conditions that may relate to serious morbidity induced from tissue-specific hypothyroidism include age-dependent dementia [108], or importantly, multiple sclerosis [109].

**Figure 2:**
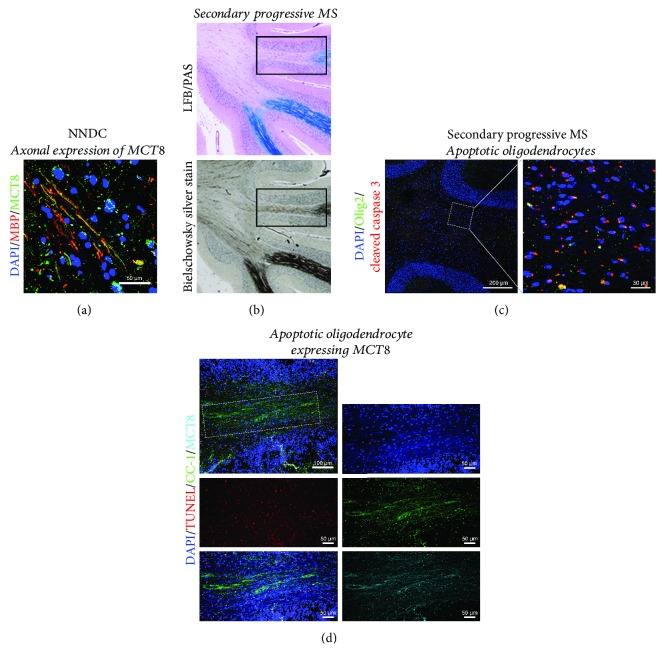
The expression of MCT8 in human oligodendrocytes and its altered expression pattern in secondary progressive MS. Immunofluorescent images illustrating (a) the axonal and oligodendroglial expression of MCT8 in nonneurological disease control (NNDC) brain tissue. (b) Secondary progressive MS cerebellar white matter stained with Luxol fast blue with periodic acid Schiff (LFB/PAS) demonstrating chronic demyelinated lesions (light purple in the center) and with Bielschowsky silver stain demonstrating axonal loss and degeneration. Immunofluorescent images illustrating (c) apoptotic Olig2+ oligodendroglial lineage cells, and (d) apoptotic oligodendrocytes expressing MCT8 in secondary progressive multiple sclerosis cerebellar white matter (outlined in the box in (b)).

This raises the tantalizing possibility that impaired MCT8, causing the acute deprivation of intracellular T_3_, may lead to hypomyelination and/or OL death, commonly associated as a contributing factor in remyelination failure exhibited in pathological lesions within the MS brain (as illustrated in Figures 1 and 2). The potential existence of a deprivation of T_3_ transport into OLs during neuroinflammation-mediated demyelination, as observed in MS lesions and in its animal models, may be a consequence of a restricted intracellular T_3_ availability for oligodendroglial lineage cells, inhibiting differentiation, with subsequently failed remyelination. In fact, it has been observed that cellular hypothyroidism occurs during EAE, impacting TH-dependent cellular processes, including OPC maturation into myelinating OLs [110]. This hypothesis may provide a new investigational platform to screen for novel therapeutics that drive neuroprotection and neurorepair during progressive MS.

## 6. Current Interventions

Synthetic TH has been utilized in preclinical and clinical trials to test the therapeutic efficacy in MS-like animal models and patients with relapsing-remitting MS (RRMS), respectively [111, 112]. Despite significant data implicating TH treatment as a method of repairing the CNS after inflammation through remyelination, the trials for MS are only at the phase I tolerability stage and so we will not know the outcomes of these for some years. However, open label trials have been recently conducted in orphan diseases using TH analogs to treat conditions such as AHDS patients, and these small molecules have included 3,5-diiodothyropropionic acid (DITPA) and 3,3′,5-triiodothyroacetic acid (Triac) [113]. It has been shown that these analogs are effective at bypassing the MCT8-dependent plasma membrane transport mechanism utilized by T_3_ [95, 114–116]. The feasibility of utilizing these TH analogs to overcome the differentiation blockade of OL precursors in TH-resistant conditions will be discussed below.

### 6.1. A Novel Treatment Option for Multiple Sclerosis

It is well established that TH is a critical factor involved in promoting OL differentiation and myelination in the early brain development and also throughout the life [117]. However, in an acquired demyelinating disease, such as MS, remyelination fails to repair chronically demyelinated lesions. The continuous failure of remyelination leads to cumulative axonal degeneration, thereby causing neurological disability including motor, sensory, and cognitive dysfunction. One of the key factors involved in the failure of remyelination is stalled OPC differentiation. Hence, utilizing T_3_ as a novel treatment option for patients with MS may enhance remyelination by activating endogenous OPCs within the CNS.

The reexpression of TH receptors in OLs may be one of the key regulators of successful remyelination in the adult CNS [43]. Moreover, it has been hypothesized that increased deiodinase 3 enzymatic deactivation of T_3_ may play a role in impaired remyelination in the animal model of MS, experimental autoimmune encephalomyelitis (EAE) [118]. Data from preclinical studies suggest that the administration of T_3_ can be effective in promoting the maturation of OPCs to protect myelin and axons, thereby enhancing the remyelination process and ameliorating the clinical symptoms of neuroinflammatory-mediated demyelination in rodent models, assisting neuroprotection [36, 48]. Further *in vivo* studies have aimed at showing that T_3_ administration was effective in reversing the expression of Olig and Shh increasing the numbers of OPCs and improving remyelination in the corpus callosum of the adult mouse brain following chronic demyelination in cuprizone-fed animals [111, 112, 119]. T_3_ treatment has also been able to induce neuroprotective effects by protecting myelin integrity and ameliorating clinical symptoms in an EAE-induced nonhuman primate *C. jacchus* (marmoset) model [120]. Moreover, it has been demonstrated that thyroid signaling was altered in the EAE-induced inflammatory demyelination rat model, with this hypothyroid state impacting upon the OPC differentiation blockade and demyelinating outcome [110]. The entirety of the neuroinflammatory models of MS have derived a clear evidence-base for T_3_ mechanism-of-action leading to human trials with T_3_ as a remyelinating therapy.

Intriguingly, evidence of CNS hypothyroid states following trauma has also emerged, with a recent study demonstrating that local delivery of T_3_ to the site of an acute spinal cord injury promoted the maturation of OLs with subsequent remyelination of demyelinated axons [121]. Collectively, these data suggest that a hypothyroid state in the CNS exists during demyelination and axonal injury, advocating that exogeneous T_3_ administration may be a potential therapeutic agent to stimulate endogenous OPCs and enhance remyelination in demyelinated lesions. In fact, currently, an open-label phase 1 clinical trial (NCT02506751) is being conducted to test the safety and tolerability of a synthetic form of T_3_, liothyronine, in patients with MS. Moreover, the investigators are determined to evaluate the effect of liothyronine on the neurotrophic factors and/or inflammatory biomarkers from enrolled patients' cerebrospinal fluid (CSF).

### 6.2. Thyroid Hormone Analogs

#### 6.2.1. Diiodothyropropionic Acid (DITPA)

Diiodothyropropionic acid (DITPA) is a TH analog which acts as a thyroid receptor agonist, to potentiate the translocation of the receptor (part of the c-erbA protooncogene family of genes) to the nucleus whereby its binding occurs at specific TH response elements (TREs) [122]. The chemical structure of DITPA is similar to triiodothyronine (T_3_) except the absence of the –NH_2_ group in the acidic side chain and reduced number of iodine atoms [123]. Its similarity in chemical structure may suggest the therapeutic potential of DITPA to treat AHDS patients replenishing the hypothyroidism present in the brains of individuals living with this severe X-linked inherited disorder. DITPA was initially tested in MCT8-deficient mice showing its bioavailability in the brain regardless of the MCT8 deficiency, and without causing a thyrotoxic effect in the liver, but being effective in normalizing the peripheral hyperthyroid status and metabolism [114]. These data have highlighted the safety and tolerability of DITPA with regard to dysfunctional metabolism. Furthermore, the efficacy of DITPA was evaluated in AHDS patients in an open label-trial in an attempt to rescue the MCT8-deficiency-induced hypermetabolism [95]. In AHDS patients, elevated levels of serum T_3_ occur causing hypermetabolism in peripheral tissues, whereas reduced T_3_ within the brain establishes a hypothyroid state within the CNS [95]. Upon DITPA treatment, hyperthyroid parameters were normalized without any significant changes in hematological parameters including hemoglobin levels and red and white blood cell numbers, along with normal serum electrolytes and renal and liver tests. Hence, hypermetabolism was reduced in AHDS children [95].

It is therefore a clinical possibility that MCT8-deficiency may be treated by DITPA administration. However, it has only been reported that this treatment within AHDS patients can stabilize the physiological abnormalities present within the central and peripheral tissues. It is inevitable that prenatal treatment is required for AHDS patients due to a lack of evidence showing the improved neurological deficits. Most importantly, the exact mechanism by which DITPA acts and how DITPA treatment can recover neurological function during the course of AHDS remains to be established.

However, recently, we addressed the current knowledge gap surrounding the mechanism-of-action of DITPA upon oligodendroglial lineage cells [87]. Specifically, our group demonstrated the effect of DITPA in hESC-derived OLs, upon acute downregulation of MCT8 [87]. Our data showed that MCT8 deficiency in human OLs potentiated cell death, which could be rescued by DITPA administration [87]. Although the metabolic parameters of the cells following DITPA stimulation are still unknown, collectively, the current data suggest that it may enhance myelination and should be reviewed for clinical trial assessment in AHDS patients, in particular if early intervention strategies at either the prenatal period or upon birth can be achieved.

#### 6.2.2. Triiodothyroacetic Acid (Triac)

Triiodothyroacetic acid (Triac) is a naturally occurring metabolite of TH in humans, which is present in the circulation at a 50-fold lower concentration than T_3_ [124, 125]. Triac particularly binds to transthyretin (TTR) and has the estimated plasma half-life of 6 h, which is cleared rapidly from the circulation despite its high affinity for plasma binding proteins [126]. It has been shown that Triac has similar affinity for TR*α*1 binding as T_3_, however, 3-6-fold higher affinity for TR*β* than T_3_, resulting in greater transcriptional activation within transfected cells [127, 128]. This leads to the conclusion that Triac may preferentially act through TR*β* isoform, which is supported by a lower EC_50_ value for TR*β* compared to TR*α*1-mediated transcriptional activation [129].

The therapeutic potential of Triac was first suggested to treat patients exhibiting resistance to thyroid hormone syndrome that clinically have high serum TH levels. The safety and tolerability of Triac has been validated by treating RTH patients [130]. It has been shown that Triac binds to the same TH receptors as T_3_, and the cellular uptake of Triac is MCT8-, MCT10-, or OATP1C1-independent [115, 131, 132]. An *in vitro* study has shown that Purkinje neurons were differentiated in the presence of either Triac or T_3_ independent of the presence or absence of MCT8 [131]. Further *in vivo* studies in MCT8-KO mice have shown the uptake of Triac in the brain with concomitant lowering of T_3_ and T_4_ levels in the serum thereby lowering the peripheral hyperthyroid status [115, 133]. Moreover, the effectiveness of Triac was tested in MCT8-OATP1C1 double knockout mice, showing profound improvement in cerebellar development and cortical myelination [115, 134]. These *in vivo* and *in vitro* studies prompted investigative studies into Triac as a therapeutic candidate to treat AHDS. Currently, a phase 1 clinical trial is being conducted to test the safety and efficacy of Triac in patients with AHDS (NCT02060474) by reducing the toxicity of high T_3_ levels and restoring the euthyroid status in the brain. Furthermore, a phase II clinical trial has been proposed to focus on the effect of Triac upon neurocognitive development (NCT02396459).

## 7. Conclusion

OLs provide structural and metabolic support to neurons in order to protect the dynamic axoglial unit in the CNS. They produce insulating membranes, known as myelin, wrapping around axons to ensure that the neuronal impulses are transmitted in an efficient manner. However, a pathological environment instigated by dysfunctional genetics and/or acquired inflammation may instigate the denudement of axons (demyelination) with disintegration of the axoglial unit, leading to profound axonal degeneration. One of the central facets of remyelination failure, as manifested in MS, is the stalled OPC differentiation possibly due to a lack of trophic support provided by TH. In fact, TH is required for the dynamic control of myelination throughout life in order to support the survival and differentiation of OLs. The hypothyroid state within the CNS may cause a significant cognitive dysfunction. Dysfunction within TH transporters in the CNS, such as MCT8, restricts the intracellular T_3_ level, causing a hypothyroid state in the CNS. Based on the collective preclinical data, THs can rescue OPC differentiation blockade to enhance their remyelinating potential. Currently, T_3_ is being tested for its tolerability and safety in patients with MS; however, TH analogs, such as DITPA or Triac may be better suited to overcome the hypothyroid status in the CNS under TH-deprived conditions due to overt inflammation-dependent changes to TH transporters upon oligodendroglial lineage cells. However, more research is required to understand the metabolism of these analogs within the CNS. Such studies will lead us to the translation of these analogs and potentially more sophisticated therapeutic strategies to overcome the differentiation blockade of OPCs in TH-deprived conditions.
